# A randomised controlled trial to compare a range of commercial or primary care led weight reduction programmes with a minimal intervention control for weight loss in obesity: the Lighten Up trial

**DOI:** 10.1186/1471-2458-10-439

**Published:** 2010-07-27

**Authors:** Kate Jolly, Amanda Daley, Peymane Adab, Amanda Lewis, John Denley, Jane Beach, Paul Aveyard

**Affiliations:** 1 School of Health & Population Sciences, University of Birmingham, Birmingham B15 2TT, UK; 2 School of Sport and Exercise Sciences, University of Birmingham, Birmingham B15 2TT, UK; 3 NHS South Birmingham, 6th Floor, Triplex House, Eckersall Road, Kings Norton, Birmingham B38 5SR, UK

## Abstract

**Background:**

Developed countries are facing a huge rise in the prevalence of obesity and its associated chronic medical problems. In the UK Primary Care Trusts are charged with addressing this in the populations they serve, but evidence about the most effective ways of delivering services is not available. The aim of this study is to determine the effectiveness of a range of weight loss programmes for obese patients in primary care and to determine the characteristics of patients who respond to an invitation to a free weight management programme.

**Methods/Design:**

Lighten Up is a randomised controlled trial comparing a range of 12-week commercial and NHS weight reduction programmes with a comparator group who are provided with 12 vouchers enabling free entrance to a local leisure centre. The weight reduction programmes are: (i) Weight Watchers, (ii) Slimming World, (iii) Rosemary Conley, (iv) a group-based dietetics-led programme (Size Down), (v) general practice one-to-one counselling, (vi) pharmacy-led one-to-one counselling, (vii) choice of any of the 6 programmes. People with obesity or overweight with a co-morbid disorder are invited to take part by a letter from their general practitioner. The sample size is 740 participants.

The primary outcome is weight loss at programme-end (3 months). Secondary outcomes are weight-loss at one year, self-reported physical activity at 3 and 12 months follow-up and percentage weight-loss at 3 months and one year.

**Discussion:**

This trial will provide evidence about the effectiveness of a range of different weight management programmes in a primary care population.

**Trial registration:**

Current Controlled Trials ISRCTN25072883

## Background

In the UK the rates of obesity have more than doubled in the last 25 years, and being overweight has become the norm for adults [1]. In 2003/2004, the mean body mass index (BMI) of men and women in the UK general population was 27 kg/m^2^, outside the healthy range of 18.5-25 kg/m^2 ^[2]. Health Survey for England 2008 data showed that nearly a quarter of men (24%) and women (25%) were obese [3], as defined by the World Health Organisation (WHO) criteria of a BMI ≥ 30 [4]. In addition, levels of physical activity were very low with only 6% of men and 4% of women meeting the government's current recommendations for physical activity [3]. A number of chronic medical conditions are associated with overweight and obesity, including type 2 diabetes, hypertension, coronary heart disease and stroke, metabolic syndrome, osteoarthritis and various cancers [2].

Health benefits have been reported with modest weight loss of 5-10% from lifestyle interventions, with a reduction in progression to diabetes of up to 58% over 4 years [5,6]. It is essential that we identify the best ways to achieve and sustain such weight loss in the general population. Douketis and colleagues [7] undertook a systematic review of controlled trials of weight loss interventions that had a follow-up of at least 2 years. In the trials that reported the outcomes of dietary and lifestyle therapy, a mean (sd) weight loss of 3.5 (+/- 2.4) kg was reported after 2-3 years follow-up. However, 9 of the 16 trials reported loss to follow-up rates ranging from 31-66% and 14 trials only reported the outcomes of study completers, thus the effect of weight loss interventions were probably over-estimated. Systematic reviews have reported that combined diet and exercise interventions result in a greater weight loss than dietary interventions alone, both in the short and longer term [8,9]. Curioni and colleagues reported the outcomes at 1 year or more from 33 randomised controlled trials (RCT) of diet, exercise, or diet and exercise [9]. Mean weight loss in diet-only trials was 4.5 +/- 11.3 kg, compared to 6.7 +/- 8.3 kg in the trials of combined diet and exercise (p = 0.063). A systematic review of trials from the major commercial and self-help weight loss programmes in the USA concluded that much of the evidence was sub-optimal, with many lacking evidence, poor quality design and with high attrition rates [10].

Much of the research on the effectiveness of weight loss interventions has been undertaken in the USA [8]. In the UK, the effectiveness of four commercial weight loss programmes was evaluated in a publicly funded randomised controlled trial [11]. This reported that all four diets (Dr Atkins' new diet revolution, Slim-Fast plan, Weight Watchers pure points programme and Rosemary Conley's eat yourself slim diet and fitness plan) resulted in clinically significant weight loss (average 5.9 kg) over the six months of follow-up. However, the trial excluded people with chronic medical conditions, such as coronary heart disease, had an upper age limit of 65 years and obtained its participants via advertising. This approach is likely to result in a sample that is not typical of the population trying to lose weight and targeted by primary care. By 12 months, follow-up was only 54% and many had changed diet programme. Weight loss at 12 months in those followed-up ranged from 9.0-10.9 kg in the groups to which they were originally allocated.

The UK Government White paper, 'Choosing Health', identified inadequate provision of services for obesity [12]. This may have stimulated development of new services because a survey of 344 primary care organisations in 2004 found that 51% had set up weight management services in primary care [13]. A feasibility study of a referral service to a commercial weight management partner reported acceptable attrition rates and weight loss in participants [14]. A primary care-based programme for obesity management in the UK, the Counterweight Programme, reported a clinically significant weight loss of 5% or more in 30% of attendees at 12 months [15-17]. A small pilot trial of a 12 week nurse-led programme in general practice reported a third of the intervention group achieving at least a 5% reduction in body weight compared to 20% in the usual care arm [18]. However the effectiveness of NHS weight loss services have not been compared with commercial providers.

This research protocol was developed in response to a need for South Birmingham Primary Care Trust (PCT) to develop services for the management of obesity. The PCT had identified that there were insufficient weight management services available in the National Health Service (NHS) and wished to develop these. As part of this, general practitioners (GPs) had been contracted to record height and weight of their patients. One option was for GP practices or NHS pharmacies to provide weight management services, for which the PCT would reimburse these practitioners. However, in the UK, there are many well established commercial companies that provide weight management services that are widely recognised and commonly used by those seeking help to lose weight. The PCT wished to contract with services that would have a high reach, good acceptability, and showed evidence of effectiveness and cost-effectiveness. Thus the aim of this trial was to determine the effectiveness of a range of NHS and commercial weight loss programmes in an unselected primary care population and to determine the characteristics of people responding to an unsolicited letter of invitation from their general practitioner.

## Methods/Design

The study design is a randomised controlled trial with patients individually allocated to one of seven weight loss programmes: (1) Weight Watchers, (2) Slimming World, (3) Rosemary Conley (4) NHS group weight loss programme (Size Down), (5) general practice one-to-one support, (6) one-to-one pharmacist support and (7) choice of intervention; with a minimal intervention control group provided with 12 vouchers enabling free entrance to a local leisure centre.

### Population

Eligible participants are registered with general practices in South Birmingham Primary Care Trust, aged ≥ 18 years, with a raised BMI recorded within their primary care notes within the previous 15 months. The BMI threshold for invitation is that which makes them eligible for primary care obesity management services within the NHS and varies according to ethnic group and the presence or absence of co-morbidities. The threshold for invitation for people with no obesity-related comorbidity is a BMI of ≥ 30 Kg/m^2 ^in any ethnic group except South Asian and BMI ≥25 Kg/m^2 ^in South Asians. For people with obesity related comorbidity the threshold for invitation is a BMI ≥ 28 Kg/m^2 ^for all ethnicities apart from South Asians, for whom it is BMI ≥ 23 Kg/m^2^.

Their GP has to confirm that they have no medical contra-indications for any of the intervention programmes. Patients are excluded if they are unable to understand English, are pregnant or are unwilling to be randomised.

Recruitment commenced in January 2009. Each general practice identifies eligible patients using a search of the computerised clinical record and invites them by standard letter to participate in the weight loss trial. Practices are asked to exclude patients with serious co-morbidities for whom weight loss programmes were unfeasible or inappropriate, for example those with terminal illness. The invitation letter contains information about the trial and a toll-free telephone number for a call centre managing the recruitment and randomisation. The nurses at the call centre provide more information to patients about the trial, answer questions, collect baseline information, take verbal consent and randomise patients to the trial arms. The call centre books participants into their first treatment session and sends confirmation, along with verification of consent and information on how to withdraw from the trial if they change their minds. Participants allocated to the general practice, pharmacy or minimal exercise intervention (comparator) arms are sent details about how to arrange their first session.

The randomisation sequence was prepared by an independent statistician and to ensure blinding, each allocation was placed in an opaque, consecutively numbered envelope. These are used in order by the call centre staff, who record the number of the envelope and time of randomisation to enable the study team to check these are used in the correct order.

Following the first programme session, the call centre telephones the participants to check whether they attended and rebook appointments if necessary. If participants have not attended and have no wish to do so, nurses record the reasons for this.

### Interventions

The content and delivery of the interventions are described in the Table 1. In addition the interventions have been classified according to Abraham and Michie's taxonomy of behaviour change techniques [19] by extracting the elements of the interventions from written manuals and materials provided by the programmes.

**Table 1:** Characteristics of weight management programmes

	Weight Watchers	Slimming World	Rosemary Conley	NHS Size Down	General practice/pharmacy
Professional background of therapist	Successful group members selected through interview process. No formal qualifications required prior to training and selection.	Successful group members selected through interview process. No formal qualifications required prior to training and selection.	Varied, may be successful slimmers. No formal qualifications required prior to training and selection.	Food advisors recruited from local community. No formal qualifications required prior to training and selection.	GP: practice nurse or general practitioner Pharmacy: pharmacist

Training of therapist	4 visits to meetings to observe and deliver elements, admin workshop; 3 day residential workshop; Further 4 meetings delivering practical elements, one admin workshop.	4 day foundation training course; 4 advanced training courses.	OCR Exercise to music training. Certificate in applied nutrition and weight management. Business management and marketing. Attendance at annual training conferences and convention.	NVQ level 3, 12 × 2.5 hour training sessions from dieticians and nutritionists. 18 assessments.	2 day adult weight management course.

Assessment of therapist's competence	Assessed by area manager, who has observed all training sessions apart from 3-day workshop.	Completion of training course and 4 diploma exams. Observations at 1^st ^and 6^th ^sessions (minimum); twice yearly development visits by manager.	Theoretical papers and practical assessment by independent body. Assessed taking class once started. Annual business review.	Observed running session prior to passing the course.	Observed during the training course by leader e.g. Q&A, mock-interviewing.

Fidelity checking of intervention	Regular observations by area service manager. Buddy system during first year.	Weekly planning and training phone call with manager; regular observations of groups. Monitoring of retention to group, weight loss achieved.	Mystery shoppers used. Random checks.	Monthly supervision meetings.	None.

**Programme Characteristics**	**Weight Watchers**	**Slimming World**	**Rosemary Conley**	**NHS Size Down**	**General practice/pharmacy**

Group or individual	Group. 1-2-1 for new members and when weighed. Group talk from leader with discussion.	Group. 1-2-1 when weighed. Group used to share progress and lapses and to find and share solutions.	Group. 1-2-1 when weighed and to establish calorie allowance. Also, additional support available via email/telephone 1-2-1.	Group. Group used to share progress and lapses and to find and share solutions. All group members start in same week and progress as a closed group.	Individual

Duration of sessions, frequency, programme length and setting	1 hour sessions. 12 weekly sessions provided as part of Lighten Up. Community based venues.	1 1/2 hour sessions. 12 weekly sessions provided as part of Lighten Up. Access to website, magazines and 1-2-1 telephone support from consultant or other members. Community based venues.	1 1/2 hours 12 weekly sessions provided as part of Lighten Up. Community based venues.	2 hour duration; groups weekly × 6 weeks; drop-in at 9 and 12 weeks. Community based venues. All group members start in same week and progress as a closed group.	1^st ^session 30 minutes, follow up sessions 15-20 minutes 12 weekly sessions provided as part of Lighten Up - although may not have taken place weekly in all cases. In surgery or pharmacy.

Content of sessions	Core programme material delivered over 5-weeks: food points system (based on age, gender, height, weight & activity), beating hunger, taking more physical activity, eating out and keeping motivated. Other sessions delivered to whole group cover recipes, health and nutrition and keeping active.	Encouraged to eat mainly low energy dense foods to achieve satiety, plus some extras rich in calcium and fibre, with controlled amounts of high energy dense foods.	Weight loss and improved diet, fitness and improvement of physical condition, motivation and self esteem, use of group support. Use of portion pots. Motivational video.	Managing behaviour around food and relapse prevention; eatwell plate [25]; nutrition information; interactive style used. Planning strategies to deal with lapses into previous dietary behaviours.	Sessions client led and based around a problem solving approach. Weight and dieting history; exploration of goals & expectations of patients; eatwell plate; setting goals to reduce calorie intake & increase physical activity. Planning strategies to deal with challenging situations. Use of food diaries. Maintaining weight loss

Weight-loss goal	0.5 to 1 kg per week (plan aims for 500 kcals deficit/day)	Set by individual.	Staged goals: either 1-1.5 kgs per week with goal of (1 stone) loss or 0.5-1 kg per week with 3.2 kg (7 lb) initial goal.	Participants told they can lose 2% of body weight in 12 weeks	5-10% of starting body weight, at a rate of 0.5-1 kg per week over 3-6 months, followed by maintenance

Relative emphasis on diet and exercise	Diet>exercise. Physical activity encouraged, objective to gradually build up to 10,000 steps daily.	Diet>exercise. Physical activity encouraged, with gradual build up to 30 mins moderately intense activity 5 days a week.	Diet=exercise 45 mins (of 11/2 hour sessions devoted to optional exercise class. Extra exercise sessions may be offered for additional fee.	Diet>activity, but time spent on benefits of physical activity, setting goals and finding activities to fit into life.	Diet=exercise Aim to slowly increase activity levels to achieve 30 minutes of moderate activity, 5 days each week;

Intervention theoretical background	Not stated	Transactional analysis, motivational interviewing; awareness of Ego States.	Not stated	Process of change [26] (Prochaska & diClemente)	Stages of change; motivational interviewing

Predominant behavioural change techniques used	Stages of change, food and activity diaries, goal setting and evaluation of progress. Rewards for every 3.2 kg (7 lbs) lost, 5% and 10% of body weight.	Weekly weighing; group support, group praise for weight loss, new decisions and continued commitment even in absence of weight loss. Awards for 3.2 kg (7 lbs) lost and loss of 10% of body weight. Individual support if needed using self-monitoring of food and emotions, for and against evaluations, visualisation techniques, personal eating plans.	Role modelling, group support. Visualisation and reframing to support behaviour change. Rewards for slimmers who maintain weight or lose, slimmer of the week and certificates for 3.2 and 6.35 kg (7 lb and 1stone) milestones.	Goal setting; stages of change; self-monitoring via food diary.	Goal setting; self-monitoring via food diaries, hunger scale, waist measurements and physical activity. Resources to provide as homework to then discuss in next session or act as personal reflection. Encouraged to make rewards to self for success.

Behavioural change techniques used (Michie categories*)	1, 4, 11, 12, 13, 14, 7, 14, 19	1, 8, 10, 11, 13, 5, 7, 23, 14, 19, 25	1, 6, 8, 4, 12, 13, 14, 19	1, 6, 8, 4, 10, 11, 12, 5, 23, 18, 26	1, 4, 12, 13, 5, 23,25, 26

*EDUCATIONAL ACTIVITIES: 1. Provide general information on behaviour-health link; 2. Provide information on consequences; 3. Provide information about others' approval; 6. Provide general encouragement; 8. Provide instruction; 9. Model/Demonstrate the behaviour.

SELF-MONITORING: 4. Prompt intention formation; 10. Prompt specific goal setting; 11. Prompt review of behavioural goals; 12. Prompt self-monitoring of behaviour; 13. Provide feedback on performance.

INTENTION FORMATION AND PLANNING: 5. Prompt barrier Identification; 7. Set graded tasks; 23. Relapse prevention.

BEHAVIOURAL TECHNIQUES TO SUPPORT CHANGE: 14. Provide contingent rewards; 15. Teach to use prompts/cues; 16. Agree behavioural contract; 17. Prompt practice; 18. Use of follow up prompts; 19. Provide opportunities for social comparison; 20. Plan social support/social change; 21. Prompt identification as role model/position advocate; 22. Prompt Self talk.

SPECIFIC OTHER TECHNIQUES: 24. Stress management; 25. Motivational interviewing; 26. Time management.

The participants allocated to the commercial operators Weight Watchers [20], Slimming World [21] and Rosemary Conley [22] have a choice of programme locations and times. Commercially, these programmes run continuously, with no set number of sessions and no fixed starting date. People who are not participating in the trial pay fees to attend these programmes and these people attend alongside those in the trial, for whom treatment is free. Each programme is provided in accordance with the respective organisations' guidance and the group leaders are trained by the organisations. The vouchers provided to trial participants exempts them from payments for the first 12 weeks of the programme. Thereafter, if participants wish to continue attending sessions they are required to pay the appropriate fees to the service provider. The Size Down Programme is an NHS group-based programme in which all members of the group commence together and follow a prescribed course of sessions. It takes place in various community venues. Participants randomised to the general practice or pharmacy arms attend one-to-one sessions in the general practice or pharmacy. Appointments are made at a time mutually convenient to the participant and the nurse/pharmacist.

In the choice arm, participants are able to choose from any of the intervention programmes described above.

Participants allocated to the comparator group are sent vouchers for 12 free sessions to attend a local authority run leisure centre (typically including an exercise room, swimming pool, and other facilities such as squash or badminton courts). Participants are not given an appointment to attend and are given no dietary or individual physical activity advice and support. It is acknowledged that weight loss can occur with exercise-only, but effects are modest [8,23].

### Data collection

#### Baseline data

Data collected at baseline by the call centre, prior to randomisation includes: demographic data, information on previous use of weight loss programmes including commercial services, current physical activity levels (using the International Physical Activity Questionnaire-short form (IPAQ-short)) [24] and use of weight loss medication. Patients allocated to the choice arm are asked about their reasons for choosing their programme.

When participants attend their first weight-loss session in the six interventions, the leader/counsellor measures participants' height and weight. Scales are validated by the research team using standardised weights, unless evidence of recent independent validation is provided. The commercial providers often use self-reported height, so this will be re-measured at follow-up by the blinded assessor. People in the comparator control group and people who are randomised but who do not attend their allocated programme are contacted and a researcher makes an appointment to measure height and weight.

During the 12-week programmes the service providers record weights on each visit. The comparator group are weighed at baseline only.

#### Outcome assessment

The primary outcome is weight loss at three months follow-up. The secondary outcomes are self-reported physical activity using the IPAQ-Short version, weight loss at one year and percentage weight loss at 3 months and one year.

At three months after programme start (programme end) the service providers weigh participants. Participants who are no longer attending their allocated programme are contacted and offered follow-up at home or another convenient location. If participants decline to be followed-up in person, they are asked to provide a self-reported weight, which is recorded as self-report. In addition, the IPAQ-short is administered by phone and participants who have dropped out of their allocated programme are sent an open-ended question asking for their views about the weight loss programme to which they were allocated.

**Figure 1:**
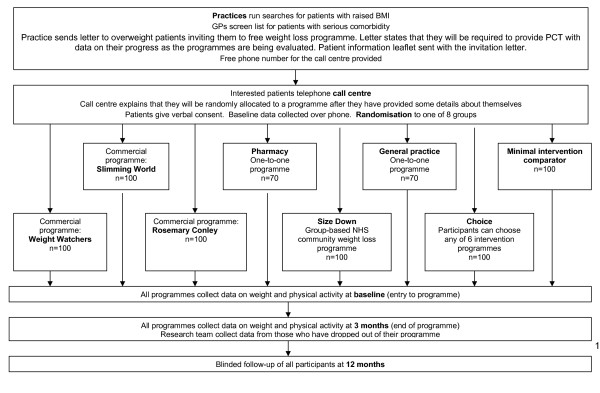
**Patient flow through trial**.

The final outcome assessment takes place one year after randomisation. In addition to assessment of their BMI, participants are asked to complete the IPAQ-Short, their opinion of the service and whether they have tried any other weight loss programmes or strategies over the course of the year. Participants are invited to attend their general practice for a brief appointment, where the practice nurse or a health trainer who is blind to the allocation arm, undertake follow up measures. Participants unable to attend their general practice will be offered home visits for measurements. If height has not been measured previously, it is assessed using a Leicester height measure. Participant flow through the trial is shown in figure 1.

### Sample size

A sample size calculation based on detecting a 2 kg difference in weight loss at the 12 week follow-up between any of the planned interventions and comparator group, with 90% power and 5% significance level found that 70 participants randomised to each group will be sufficient. This estimate is based on a standard deviation of 3.2 kg [11,14] and allows for a 20% dropout rate. To enable a more realistic drop-out rate and to get greater precision, 100 participants were allocated to each arm. However, not many general practices or pharmacies were prepared to offer treatment themselves and training places were limited, so that only 70 will be allocated to these arms. A total of 740 participants will be randomized.

### Planned analysis

Both between and within group analyses for primary and secondary outcomes at programme end and 12 months will be performed according to intention to treat. Participants in whom weight at follow up is not available will be assumed to have their baseline weight. However, sensitivity analysis will also be undertaken, assuming follow up weight to be the last recorded weight.

As the outcomes are all measured on a continuous scale (weight loss, percentage weight loss, self-reported physical activity), differences between intervention group and the comparison (minimal intervention) group will be investigated using least squares linear regression. Between group analyses will be expressed as both unadjusted and adjusted differences (corrected for baseline score and covariates of age, gender, ethnicity and educational level). A secondary analysis will compare the outcomes of the commercial weight loss programmes (Weight Watchers, Slimming World and Rosemary Conley) with the primary care programmes (general practice and pharmacy-based interventions). Data on choice of programme, number of sessions attended and reasons for drop-out will be presented using descriptive statistics.

To determine whether the service reaches those most in need, the age, gender, and deprivation score (derived from the postcode of residence) of those who were randomised will be compared to those who received the letter but did not respond or declined to be randomised.

Responses to open-ended questions about experience of the service and reasons for dropping out of their allocated programme will be categorised and presented descriptively.

### Costs

An NHS perspective will be taken in the cost analysis. The price to the PCT of each programme will be used. The cost of the call center which co-ordinated the service will be calculated as an average per person, based on the numbers of staff employed over a 12 month period and clients who used the service over this time period. The mean cost per kg of weight lost will be determined for each programme.

### Ethical approval

Ethical approval was granted by South Birmingham Research Ethics Committee (08/H1207/331).

## Discussion

A quarter of the UK population and a similar high proportion in other countries are obese. If primary care clinicians were to manage this disorder, it would potentially mean a large proportion of their time being devoted to it. Commercial weight loss organizations have a large network of treatment services that provide a cheaper alternative to primary care services. However, the comparative effectiveness and the cost-effectiveness is unknown. This trial aims to provide these data.

One important aspect to this work is that it is a weight loss trial recruiting in primary care among those who have not specifically sought treatment. Many published weight loss intervention trials treat patients in specialist obesity clinics. These clinics provide treatment for only a minority of all people who need it. In particular, in this study, we will be able to examine uptake of obesity management services in response to an invitation. We are not aware of any other study that has examined uptake in a defined population in this way.

Robust clinical trial results are not available for all the commercial providers in this trial and for few primary care led programmes, so this research will extend knowledge about the efficacy of commercial and community weight loss programmes. In addition our participants were responders to an invitation from their general practitioner, whereas many other trials have recruited volunteers, who are likely to be more motivated to lose weight. This trial will inform decisions about which weight loss programmes might make useful clinical contributions for patients with obesity and which might have useful contributions to reducing the public health toll of obesity.

## Abbreviations

BMI: body mass index; GP: general practice; IPAQ: international physical activity Questionnaire; UK: United Kingdom.

## Competing interests

The authors declare that they have no competing interests.

## Authors' contributions

KJ, AD, PA, JD, JB and PA designed the study and wrote the initial protocol. KJ, AL, AD, PA, JD, JB and PA are on the trial management committee. AL co-ordinated the study with supervision from KJ. KJ drafted the manuscript with contributions from the other authors. All authors read and approved the final manuscript.

## Pre-publication history

The pre-publication history for this paper can be accessed here:

http://www.biomedcentral.com/1471-2458/10/439/prepub
